# Understanding the misophonic experience: a mixed method study

**DOI:** 10.3389/fpsyg.2025.1493676

**Published:** 2025-02-05

**Authors:** Yesim Ozuer, Rilana Cima, Elke Kestens, Ilse Van Diest

**Affiliations:** ^1^Research Group Health Psychology, Faculty of Psychology and Educational Sciences, KU Leuven, Leuven, Belgium; ^2^Adelante, Centre for Expertise in Rehabilitation and Audiology, Hoensbroek, Netherlands; ^3^Department of Clinical Psychological Science, Maastricht University, Maastrich, Netherlands

**Keywords:** misophonia, thematic analysis, emotional representations, meaning information, defensive anger, factor analysis

## Abstract

Misophonia is a poorly understood condition in which intense distress is experienced in response to mostly orofacial stimuli. To better understand why specifically anger and disgust seem to characterize this distress, we investigated meanings conveyed by misophonic trigger stimuli in two studies. Study 1 explored these meanings and emotions in two small focus groups (*n* = 3, *n* = 5) of misophonia sufferers. Four meaning—themes were generated based using reflexive thematic analysis: “intrusion,” “violation,” “offense,” and “lack of autonomy.” Also, four emotional reaction themes were constructed: “anger/defensive rage,” “disgust,” “fear,” and “safety behaviors.” Study 2 aimed to corroborate the findings of Study 1 in a large, independent sample. To this end, misophonia symptom severity was assessed in 431 young adults using the Amsterdam Misophonia Scale (A-Miso-S). Participants rated the extent to which the meanings and reactions identified in Study 1 matched their experiences with prototypical misophonic trigger stimuli. The meanings showed a positive, moderate correlation with misophonia symptom severity and accounted for 35.15% of the variance in A-Miso-S scores. An exploratory factor analysis identified two factors explaining 50% of the variance in the meanings and reactions. Factor 1, “Avoidance of intrusive/disgusting stimuli” had high and unique loadings on avoidance, intrusion, and disgust. Factor 2, “Autonomy/Violation,” had high and unique loadings on violation, lack of autonomy, offense, and defensive rage. These findings suggest that the meanings of intrusion, violation, and lack of autonomy are inherent to the misophonic experience, with potential implications for treatment strategies.

## 1 Introduction

Misophonia is a disorder characterized by an excessive negative emotional response or decreased tolerance toward—most typically— orofacial human sounds related to breathing and eating (e.g., Bernstein et al., 2013; Brout et al., 2018; Dozier, 2015a; Edelstein et al., 2013; Jastreboff and Jastreboff, 2002; Jastreboff and Jastreboff, 2015; Siepsiak et al., 2020; Hansen et al., 2021; Hansen et al., 2022; Swedo et al., 2022). Consensus is growing, however, that trigger stimuli can involve other sounds (typing, ball bouncing, walking in heels; e.g., Hansen et al., 2021) and modalities as well (visual, kinesthetic; e.g., Claiborn et al., 2020; Ferrer-Torres and Giménez-Llort, 2022; Jager et al., 2020a). The core emotional reactions toward trigger stimuli seem to consist of anger and disgust—whether moral or visceral—rather than anxiety (Cavanna and Seri, 2015; Dibb and Golding, 2022; Edelstein et al., 2013; Palumbo et al., 2018; Iskander et al., 2023; Norena, 2024).

Although misophonia is currently not recognized as a diagnostic separate entity in the DSM-5, it associated with a low quality of life, and poses also a burden on those who live with misophonia sufferers (Dibb and Golding, 2022; Guzick et al., 2023; Jager et al., 2020a). Current literature suggests that the onset of misophonia is situated typically, but not exclusively, in childhood or adolescence (Dozier, 2015a; Guzick et al., 2023; Johnson et al., 2013; Kumar et al., 2017; Rouw and Erfanian, 2018; Potgieter et al., 2019; Siepsiak et al., 2023), with initial complaints of misophonia being experienced on average at the age of 13 (Jager et al., 2020a) or as early as age of 9 (Dixon et al., 2024). However, since there are no general population-based studies on this topic, and findings of both latter studies are based on retrospective reports from adults with misophonia symptoms, the age of onset remains unclear.

Recent studies with undergraduate student populations have provided growing insights into the prevalence of misophonia across various cultures. Prevalence rates were found to be 19.9% in the USA using the Misophonia Questionnaire (MQ) (Wu et al., 2014) and 15.85% among Indian college students responding to the Amsterdam Misophonia Scale (A-Miso-S) (Schröder et al., 2013; Patel et al., 2023). General population studies using the A-Miso-S (Schröder et al., 2013) reported prevalence rates ranging from 5.9% in a German sample (Jakubovski et al., 2022) to 49.1% in the UK, although only 0.3% reported very extreme symptoms (Naylor et al., 2020). A recent study using multiple assessments, such as the S-Five, MQ, A-Miso-S, and a diagnostic interview, estimated an 18% prevalence in the general population in the UK (Vitoratou et al., 2023). Notably, a recent population-based study conducted in the USA estimated a prevalence of 4.6% at clinical levels and 78.5% for sensitivity to misophonic sounds in a nationally representative sample of adults (Dixon et al., 2024). Despite these insights, as noted by Möllmann et al. (2023) and Wu et al. (2014), a robust prevalence estimate is hard to make due to the lack of consensus on diagnostic methods (Ferrer-Torres and Giménez-Llort, 2022).

Until recently, research on misophonia primarily centered on its definition, assessment, treatment, and comorbidities (e.g., Baguley and McFerran, 2011; Jastreboff and Jastreboff, 2002; Schröder et al., 2013; Schröder et al., 2017; Rouw and Erfanian, 2018; Rosenthal et al., 2023). The complex nature of misophonia is increasingly recognized and evident from its apparent emotional underpinnings, such as anger, anxiety, and disgust (McMahon et al., 2024), its transdiagnostic nature and comorbidity with conditions such as mood, anxiety, and obsessive-compulsive disorders (Guzick et al., 2023; Herdi and Yıldırım, 2024), as well as the diversity of its triggers. Given this complexity, it has been recognized that the field and especially the treatment of misophonia could benefit from a deeper understanding of the nature and origin of the condition (e.g., Jager et al., 2020a; Kumar et al., 2017; Mattson et al., 2023; Taylor, 2017).

Etiological mechanisms of misophonia have been studied from various perspectives, including neuroscience, auditory science, and psychology (e.g., Hansen et al., 2021; Kumar et al., 2021; Hansen et al., 2022; Swedo et al., 2022; Rosenthal et al., 2023; Berger et al., 2024; Neacsiu et al., 2024). In this paper, we specifically focus on the psychological aspects of the condition to deepen our understanding of the misophonic experience. Here, we aim to focus specifically on the psychological aspects of the condition that may help understanding the misophonic experience.

An interesting, yet open question in this respect is why, to our knowledge, anger and disgust, rather than fear or other emotions, seem to constitute the core emotional reactions to misophonic triggers. As physical properties of the sound (e.g., loudness, pitch, timbre) appear unrelated to the intensity or nature of emotional reactions (Edelstein et al., 2013), it has been hypothesized that specific meanings of the trigger stimuli are inherently related to the emotional reaction,—which can comprise changes in in conscious experience, behavior, and/or (neuro) physiological processes (Jastreboff and Jastreboff, 2015; Swedo et al., 2022). The importance of meanings in emotion formation align with the theory of constructed emotion (Barrett, 2017), and with the hypothesized nature of emotional representations in memory by Lang (1979) and Lang et al. (1980). According to the theory of constructed emotion, emotions arise from the brain’s attempt to make sense of the continuous stream of sensory information stemming from the inner (body) and outer environment. As such, emotions can be considered a meaning making process. To do so, the brain predicts visceromotor and skeletomotor actions as well as the sensory inputs that are expected to result from them. This inference is constructed from ongoing sensory input, as well as from learned or innate priors (Barrett, 2017). Following Lang’s bio-informational theory (Lang, 1979; Lang et al., 1980), the memory representation of a strong emotional experience can be considered a coherent associative network containing three types of information: stimulus, response, and meaning. In this framework, activating one element of the network (e.g., perceiving an orofacial sound) may activate the entire network, leading to an emotional experience. Whereas the prototypical misophonic trigger stimuli and the associated physiological and behavioral response patterns have been well-documented (e.g., Brout et al., 2018; Kumar et al., 2017; Möllmann et al., 2023; Swedo et al., 2022), less is known about “meaning” information in the representation of misophonic experiences. Swedo et al. (2022) highlight that misophonic responses often seem to be triggered not by the loudness of the auditory stimuli but by specific patterns or personal meanings attributed to the sounds. This aligns well with recent findings on the importance of context and social cognition in misophonia (Siepsiak et al., 2023; Berger et al., 2024) and suggests that individuals with misophonia may assign particular significance to trigger sounds that extends beyond their purely auditory characteristics.

The literature indicates that these associated meanings may vary depending on the individual’s learning history, socio-cultural environment, and socialization, as well as the person or source generating the sounds (Bernstein et al., 2013; Edelstein et al., 2013; Norena, 2024). Some case studies and other sources suggest that meanings related to disrespect, violation, injustice, and offense may characterize the misophonic experience (e.g., Cowan et al., 2021; Gregory and Foster, 2023; Gregory et al., 2024; Norena, 2024), but potential meanings have not been systematically investigated to date. Identifying the “meanings” that misophonia sufferers associate with their trigger sounds is crucial to advancing our understanding of misophonic experiences.

The current studies sought to investigate potentially common meanings associated with trigger sounds in individuals suffering from misophonia symptoms. Identifying these common meanings can be expected to enhance our understanding of the anger and disgust reactions experienced in persons with misophonia, potentially leading to more effective treatment strategies on the longer term. To uncover potential meanings associated with stimuli in individuals with misophonia, we conducted two methodologically distinct studies.

The first study consisted of two semi-structured focus groups conducted with a few persons suffering from misophonia. A thematic analysis on the focus groups’ transcripts was conducted to unveil potential meanings participants experience upon confrontation with trigger stimuli. A second study sought to validate the meanings identified in study 1. To this end, we conducted a questionnaire study with a larger, independent sample of respondents to examine the extent to which these meanings are related to the presence and severity of misophonia symptoms.

## 2 Study 1

### 2.1 Materials and methods

#### 2.1.1 Participants

Persons with symptoms of misophonia were recruited using social media (i.e., Facebook, Twitter, LinkedIn). The recruitment poster included the following information; “Looking for people who are experiencing symptoms of misophonia or have been diagnosed with misophonia by a mental health professional. We invite you to participate in our focus group sessions to improve our understanding of the condition.” Prospective participants who expressed interest via email were sent an invitation to complete an informed consent form and a two-part online questionnaire. To qualify, participants needed to meet the following criteria: (a) be over 17 years old, (b) score above 10 on the A-Miso-S (Schröder et al., 2013), and (c) be fluent in Dutch. Before proceeding to the questionnaire, participants underwent screening for hearing loss and self-reported history of tinnitus or hyperacusis. Eligible participants were then invited to participate. Eight participants were recruited, (see Table 1 for demographics) and were reimbursed with a 10 Euro online shopping voucher. The study was pre-registered on OSF (Open Science Framework; doi: 10.17605/OSF.IO/JZMYB) and approved by the ethical committee of KU Leuven University (SMEC; approval number is G-2021-3500-R2 (AMD)).

**Table 1:** Demographics of participants in both focus groups (*N* = 8) from the survey before the focus groups.

	Focus group 1	Focus group 2	Both groups
Mean age (years), range	46, 34–65	41.67, 23–58	44.38, 23–58
Female n (%)	4 (80)	2 (66.67)	6 (75)
Mean A-Miso-S (Schröder et al., 2013) score, range	12.2, 11–14	14, 10–20	12.88, 10–20

Information in this table consists of answers collected on a survey prior to the focus groups.

#### 2.1.2 Procedure

To allow maximal participation and discussion with eligible participants, 2 separate focus groups were organized at different time points, depending on the participants’ availabilities. Discussions took place in a secure online setting (i.e., Microsoft Teams) on May 2021 and on June 2021. Both focus groups (*n* = 3, *n* = 5) were moderated by the same student in clinical psychology (A3) using a focus group manual with semi-structured questions in presence of a licensed clinical psychologist (A2/A4) as second moderators, and an observer (A1). Duration of the first and second focus group sessions were 2 h and 55 min and 2 h and 10 min, respectively. Sessions were recorded with the written consent of the participants and transcribed verbatim by the moderator of the focus groups (A3).

Using a standardized manual, the focus group sessions were structured into three sections, (*i*) thinking about events in which participants felt triggered (*ii*) reflecting on reactions present during the events and (*iii*) discussion of the possible meaning of the trigger stimuli (see Standardized Manual in Supplementary material). In the first section, participants were asked to imagine a recent and memorable event in which they experienced the symptoms of misophonia and had difficulty containing their reactions. In the second section, each person was asked to describe emotional and physical experiences regarding the triggers and the emotional, behavioral and physiological reactions directed toward the source of the sound. Lastly, participants were asked to elaborate on possible meanings of the trigger sounds. To facilitate this, they were invited to think about what unwanted experiences the sounds may convey to them, and whether there are specific people they associate with these sounds. At the start of the focus groups, participants were introduced to ground rules (i.e., being respectful to each other’s opinions and feelings and not interrupting others when they are speaking), to maintain a respectful and safe environment for everyone. Participants raised their (virtual) hands when they wanted to participate in the discussion and they were given opportunity to communicate with each other as well.

##### 2.1.2.1 Questionnaires

One week prior to the focus group session, participants provided demographical information (see Table 1) and answered questions related to their misophonia (see Table 1) as well as the A-Miso-S (Schröder et al., 2013), a 6-item questionnaire based on the Yale-Brown Obsessive-Compulsive Scale (Y-BOCS; Goodman et al., 1989) adapted for misophonia by Schröder et al. (2013). The A-Miso-S (Schröder et al., 2013) allows classifying severity of misophonia complaints (0–4 subclinical, 5–9 mild, 10–14 moderate, 15–19 severe and 20–24 extreme). The A-Miso-S (Schröder et al., 2013) questionnaire assesses various aspects of misophonia experiences over the past week, including the degree of annoyance or preoccupation with trigger sounds, the extent to which daily activities were affected, the level of distress caused by misophonia sounds, efforts to divert attention from these sounds, and the degree of avoidance of triggering situations.

#### 2.1.3 Data analysis

We applied a reflexive deductive thematic analysis to the transcripts to construct overarching themes that seemed to refer to meanings conveyed by trigger stimuli, and to prototypical reactions and behaviors evoked by trigger stimuli. To this end, we identified, analyzed and reported occurrences of interest within the transcripts that represent a significant aspect of the data in relation to these questions (Braun and Clarke, 2006). Inspired by Braun and Clarke (2006) the thematic analysis involved the following 6 steps, (1) familiarizing with the data, (2) generating initial codes, (3) searching for themes, (4) reviewing themes, (5) defining and naming themes, (6) producing the report. In line with Braun and Clarke’s thematic analysis guidelines (Braun and Clarke, 2006; Braun and Clarke, 2019), we approached our data using a reflexive deductive approach. While we did not search for specific, hypothesized themes in the transcripts, the thematic analysis was clearly guided by our overarching interest in exploring the potential meanings and emotions tied to misophonia triggers. The approach taken was to prioritize deeper interpretive insights rather than descriptive summaries (see Braun and Clarke, 2022, paper, for a distinction between both approaches). Initially, 3 authors (A1, A2, A4) read the transcripts 3 times thoroughly and generated initial codes relating to the potential meanings associated with trigger sounds. Secondly, a list of potential theme-related codes, along with supporting extracts from the raw data was created. In the third step, each code was analyzed to be collated under possible overarching themes with the use of thematic maps. During the revision phase, authors independently assessed the identified themes for sufficient data support, then divided them into three sets (meanings, primary reactions, secondary reactions), forming the final thematic map. In determining the classification of a theme, the authors discussed whether the codes that create an overarching theme formed a coherent pattern and whether the final thematic map captured the essence of the data (Braun and Clarke, 2006). Finally, a list of all of the main themes was established including precise descriptions. All stages of the qualitative analyses were conducted using the Dutch transcript. Later, some of the sentences and phrases were translated to English by the authors in order to be used as examples in this manuscript.

### 2.2 Results

Seven themes generated from the thematic analysis (see Table 2), which could be further organized into 3 higher-order sets/categories. A first set of themes referred to 3 recurring meanings associated with trigger sounds (“associated meanings”): *intrusion/violation*, *offense* and *lack of autonomy*. The second set, “primary emotional reactions,” comprised themes referring to instantaneous emotional responses to trigger sounds: *anger/defensive rage* and *disgus*t. The third set, “secondary responses,” included themes referring to secondary emotions and less impulsive behaviors to deal with the anticipated or actual misophonic threatening situations: *fear*, and *safety behaviors*. Note that the term “set” is used here for descriptive purposes to conceptually organize the themes and is not a component of our thematic analysis approach.

**Table 2:** Direct quotes from the transcripts of both focus group sessions and the themes derived from the transcripts.

Theme	Direct quotes
Intrusion/Violation	“Gosh, that’s just annoying that I just cannot tolerate those sounds and they do not understand my feelings. They just do that on purpose because they know that it triggers me. That’s really annoying. Just the feeling that they do not understand me. And that they do not know what they are doing wrong.” (Participant 2)
	“I think so, Participant 4, because for example I matched perfectly with my father, not with my mother, but perfectly with my father, and yet it was him who was the source of my irritation.” (Participant 3)
	“Maybe because you grant more autonomy and intention to a human than to a pig. Yes, a pig has no other choice, it smacks and does not think about it, while a human can choose not to smack…What would a pig have against me, while another human being could have something against me. Come on, purely theoretical. A pig has nothing against you, it just does.” (Participant 4)
	“Yes. That would be the same if someone poked me with his finger, you do not do that either and that’s actually how it comes across to me with someone with a bik. Only that it is auditory instead of physical that they tell me.” (Participant 4)
	“And what I am also very concerned with, especially towards others in the misophonia group, is that every *misophone* has the right to a safe place and to a safe person. So I’ve made myself a safe place by just always walking around with earplugs, because you absolutely have to have a place where you can go and where you can be absolutely sure that I will never experience it. And a safe person, I have that too, my best friend is my safe person for me and that means: he understands it, he has it a bit himself, so yes, he understands it anyway.” (Participant 4)
	“Yes, I also think because they are contact sounds, so they go through the floors and they go through the walls, so I can hear that inside, but I experience such a moment every time: (him) I am in my safe bubble, in my home environment where I expect to be safe, to be calm and then suddenly something like this comes in and it actually breaks through that safety and that peace and basically everything you expect from a home environment and that hits you so hard that you are indeed shocked by it and you immediately that fight or flight reaction that I have. So either you become angry because you cannot flee or you want to flee, but fleeing is not an option for me, so every time it’s to anger succeeds” (Participant 5)
	“Yeah or a violation of your experiences at the moment, then we make it a bit smaller. Yes, that might be a little more accurate.” (Participant 6)
	“But to actually look for an explanation for that, perhaps from my person or something, I have never actually gone that far, but if I think about it a little further, it often has to do with the fact that it occurs, that it almost is an infringement on you, on your world of thought, on your own world.” (Participant 6)
	“And then, the immediate reaction is: I do get angry because I then have the feeling: It’s as if that person is suddenly in my apartment, so he has crossed my boundary.” (Participant 5)
	“And that that sound, well, the source is that person who just chews who does not even eat with his mouth open or something like that, hey just the chew chewing sound, that concrete mixer that keeps going, oh.” (Participant 1)
Offense	“…but if that filter is not there, and it concerns things that people do, they are very offensive can happen.” (Participant 4)
	“Yes, just that, I just do not think that’s polite. That’s distasteful, disgusting. I do not need to see what you have in your mouth” (Participant 2)
	“The feeling of this is not an education… (they are) badly brought up.” (Participant 3)
	“And I heard that once and then I thought: okay, that was dirty. I also find that very rude. But I thought okay, that’s possible one time, but yes, that person really did that repeatedly.” (Participant 7)
Lack of autonomy	“The feeling of why, why am I like this and why is not everyone like this? And then no other people?…Mainly powerlessness I think I am, yes.” (Participant 3)
	“Yes, what I really like is: I thought for a long time that I was a freak and that I was alone in the world, the only one who had that, together with my sister. And that there must have been something about our upbringing that had completely messed us up.” (Participant 4)
	“I hope that, I hope that nothing wrong will happen there because of me. But at that moment I thought: This is the only way I can do anything to stop this now because otherwise I will go crazy here, I will go crazy here and yes I had to, I had to be home too, I had to work, I had to behave normally in a work context and that just wasn’t about that noise all day long.” (Participant 5)
	“Now, what I do not have at all that Participant 5 has: I do not expect understanding from people who do not know me. Because I find my own situation so excessive, I think my behavior is simply disproportionate to the action, so I do not expect any understanding from people who do not know me. I expect a lot of understanding from people who do know me.” (Participant 6)
	“But it’s like that I do not know who said that before: If I know that I could not avoid it, that I’m stuck in a situation, then I also think, wow, what could I do here? being able to do, it gives such an overwhelming feeling.” (Participant 8)
Anger/Defensive rage	“Because my upstairs neighbor was a 98-year-old man. His TV was on maximum, I could literally do anything, I stormed upstairs several times to shout at that man, almost. So that, but and then, I hear too good, too good.” (Participant 3)
	“I even have it with, I’m going to be honest, I have two dogs. I love them, but when my dogs start licking themselves, I also feel like shooting through the ceiling.” (Participant 3)
	“Also, that one anger that aggression. Sometimes it really starts to look red before your eyes if yes, then that’s just yes, no, no button that flies around.” (Participant 5)
Disgust	“So, then I focus even harder on those sounds of the food, because all three of them are smacking the table, all three of them. And especially when mom eats yogurt, oh that’s real, oh I’m so disgusted.” (Participant 2)
	“it is also a very strong emotion that comes to the fore, so yes, disgust and stronger than that dislike even like yes really.” (Participant 6)
	“It would also explain why sounds that are not made by humans are also annoying, but you will have less disgust there.” (Participant 4)
Safety behaviors	“Yes, noise canceling or something, but you are sure that you can always go to something like that, to channel your flight behavior a bit, like that. Come on, I have it that way on purpose.” (Participant 4)
	“Social circumstances, on the train I will do the same as Participant 7, you just leave or you put in earphones. I will never sit on a train without music.” (Participant 6)
	“And with looking for tactics of what can help me now to deal with this and if, that is mainly yes, putting in earplugs, putting on white noise music.” (Participant 9)
	“Yes, I have noticed that it is better to put on headphones or put in earplugs or whatever or go outside if possible.” (Participant 5)
Fear	“…either I have to react or I have to run away, but I think there is also a part of fear. And you have, yes, the fear because you anticipate: a feeling may come, so you are already walking on eggshells. But I suspect that the moment that happens, it must have something to do with fear or something and hey, I have not quite figured out yet what it is exactly. But what I feel very strongly is: where do you get the nerve to penetrate me so much that I have to concern myself with you, that’s all.” (Participant 4)
	“Sometimes I even just plan my day knowing that at that moment there will be a noise, so I will not be in the kitchen when my neighbors also start cooking around that moment. Which is quite obsessive, which also leads to that spiral of fear and doubt and shame.” (Participant 5)

The original transcript is in Dutch, the relevant text was translated by the authors. The authors can be contacted for the original (Dutch) text. Extracts used in the results section do not appear in the table to avoid repetition.

#### 2.2.1 Associated meanings

##### 2.2.1.1 Theme 1: Intrusion/violation

This theme captured a significant aspect of the participants’ misophonia, namely; others being responsible for the aversive experience. The experience of being intruded upon or violated in privacy by others came up as a solid theme that typically triggered defensive anger. Illustrative extracts for this theme emphasized that the source of the sound was perceived as an intentional attack (see Table 2 for a direct quote from the participant regarding the source of the sound). Participants typically assumed that persons generating the sound were aware of the anger they caused, or ought to be aware. As such, the source of the sound violated the expected state of safety in the misophonia sufferer. The expected feeling of safety was derived from descriptions such as “my house” or “my personal area,” suggesting that the participant expected their boundaries to be known by the source of the sound. Related to this, lack of understanding from family or friends or the lack of respect from strangers was a common experience described by the participants, likely associated with the subjective perception that their boundaries were not respected.

The perception of intrusion/violation was associated with the person who is generating the trigger, rather than the sound itself, directing emotional reactions to the person rather than the general circumstances or the sounds. During the focus groups, examples of where intrusion and violation were experienced included a partner cutting nails and family members eating loudly at the dinner table. In each of these cases, participants mentioned that those who were making the sounds were responsible for their behavior, which led to a perception of intrusion and violation of personal space. For example, when the participant mentioned their “partner cutting nails,” they saw it as an intentional intruding behavior, given their partner’s awareness of their misophonia. For instance, a participant described the situation as an assault:

*“Yes, I think, you, I feel assaulted, is not the right word, because that would trivialize some things, of course that would trivialize real assaults, but I feel very much in my person, in my, yes in my, I do not know how to say it, actually assaulted, like yes, you are entering an area where you are not allowed to enter. Very much as if I were standing naked in front of someone without being asked, that’s what it actually comes down to. It is very unfair I feel treated unfairly.” (Participant 4).*

##### 2.2.1.2 Theme 2: offense

This theme captured the aspect of misophonia corresponding to the distress and perceived attack caused by others when there is no intentionality perceived by the misophonia sufferer. Even if the actions of the people creating the trigger sounds were not perceived as intentional attacks, there is still an offense taken by the misophonia sufferer. An offensive context can be defined as a situation in which the misophonia sufferer expects to be unintentionally violated by others (e.g., a public cafeteria). In an offensive context, the misophonia-sufferer realizes that their needs, sensitivities and expectancies may not be clear to others and those generating the offensive sounds might not have been aware of the emotional distress they cause. When describing offensive contexts, no known sources of the sound were mentioned, rather the context was aversive in nature because there might be potential sources of the sound who are unknown to the participant beforehand. It is also important to note that participants identified certain scenarios in which they felt offended because people around them fail to behave according to their rules. Participants cited noisy restaurants, trains, and cafeterias as offensive contexts and mentioned individuals in their lives who repeatedly clean their noses or create other orofacial sounds as potentially offensive. Overall, trigger stimuli produced by other persons were described as impolite and offensive, while easily evoking an intense and immediate anger or disgust reaction in misophonia sufferers. An example of this can be seen in the following testimony:

*“That’s, that’s just super loud it’s very recognizable from that one misunderstanding and yes, thinking that others should indeed simply be held up that mirror. That they see their own behavior from the outside.” (Participant 1).*

##### 2.2.1.3 Theme 3: lack of autonomy

This theme was generated frequently when participants described situations where they felt a profound lack of control over their own actions, emotions, and surroundings, coupled with a sense of not recognizing themselves. It captures experiences such as an inability to change or escape unwanted situations and the emotional barriers they could not overcome. Importantly, it is not limited to situations where a specific individual was perceived as a source of the sound who had control over the trigger stimuli, but also in instances where the misophonia sufferer felt a lack of control over their own emotions and reactions to the trigger sound, or over the situation in general. In reflecting on these experiences, we identified “loss of control” as a recurring term that contributed to a broader sense of diminished autonomy. The overarching theme “Lack of autonomy” encompasses various aspects of autonomy loss, including feelings of not recognizing oneself, feeling trapped by circumstances that dictate one’s emotional state, and a sense of misunderstanding from others. Participants expressed that they were “controlled” not only by the actions of others (e.g., feeling forced to tolerate triggering sounds in shared spaces like trains) but also by the misophonia itself, which led to self-imposed restrictions to avoid anticipated triggers.

Unlike other themes, “lack of autonomy” did not appear verbatim in the transcripts but was derived from various phrases that conveyed this broader sense of constrained agency. Participants expressed frustration and self-doubt, questioning, “Why am I like this?” and feeling as though “no one else” shared their experience. Other participants described a sense of “powerlessness,” feeling like a “freak” or that their reactions were “disproportionate” or “aberrant.” Statements such as “I am stuck in a situation” and “I’m going crazy here” illustrate the depth of their helplessness, where participants felt constrained by their own emotional responses rather than physical barriers. Along with these, the lack of control created a fundamental sense of desperation, linking directly to a diminished sense of autonomy. A participant described the feeling in the following way:

*“Why am I like this? Why is this happening to me? And what about other people? It’s a feeling, I think, or a meaning of powerlessness.” (Participant 3).*

#### 2.2.2 Primary emotional responses

##### 2.2.2.1 Theme 4: anger/defensive rage

When one experiences an intrusion or violation of one’s own space, one is motivated to protect themselves and their surroundings and this protection can express itself as rage (Siegel and Victoroff, 2009). Defensive rage is a highly reactive and impulsive response to perceived threats, marked by an abrupt escalation in sympathetic nervous system activity (Larson and Langer, 1997; Blanchard et al., 2001). Unlike predatory aggression, which is more calculated and goal-oriented, defensive rage is a rapid, reflexive response aimed at protecting oneself from immediate danger (Siegel and Victoroff, 2009). This form of aggression is often accompanied by heightened arousal, intense physiological activation (such as increased heart rate and blood pressure), and behaviors intended to repel or intimidate the perceived threat (Larson and Langer, 1997; Blanchard et al., 2001; Siegel and Victoroff, 2009). Our participants’ descriptions of their emotional responses to misophonic sounds align with this definition of defensive rage. It was most apparent when they encountered a situation in which they experience intrusion and violation. While participants describe intense emotional responses, these do not always manifest as overt aggression. As reflected in phrases like, “I also feel like shooting through the ceiling” the emotional surge often remains internal, without escalating into physical or verbal aggression. As the feeling of defensive rage is related to an urgent situation wherein one needs immediate protection of oneself, there is a lack of thinking and purposeful behavior toward the situation. Likewise, the misophonia sufferer reacts impulsively to stimuli or situations that are experienced as offensive/intrusive/devaluating to one’s autonomy. During the focus group sessions, the current theme mostly came up when the source of the sound was someone the misophonia sufferer knows well. These results were obtained from phrases of participants such as; “like a match that goes off,” “I flipped at that moment,” etc. An example of the anger experienced by a participant was phrased with the following words:

*“That’s real, I get there aggressive by. I always compare that a bit with if you know that about wild animals, that sketch where that lady suddenly does that. That’s really yes. That is the perfect wording and physically it gives me one too accelerated heart rate: my heart starts beating faster yes, that is really a purely physical reaction. And yes, really pure aggression. Yes, not physically of course because I would like to, but that’s not in me. But just verbal Hey, I get very aggressive verbally. Not in social circumstances, eh, in private circumstances eh.” (Participant 6).*

##### 2.2.2.2 Theme 5: disgust

Although defensive rage was the dominant primary emotional response for most participants, disgust toward the sound and its source was also common. Similar to defensive rage, the feeling of disgust was not observed as a reaction that required conscious mental processing, rather, the participants mentioned it mostly when they were describing events in which they encountered trigger sounds of misophonia. Interestingly, participants mostly identified the sound-generating people as “disgusting” rather than the action or the sound itself. In contrast to other themes derived from participants’ detailed descriptions of emotions and events, they directly labeled certain events and people as disgusting. Participants repetitively used phrases such as “they disgust me,” “this person disgusts me,” “I experience an unbelievably intense disgust” etc. Lastly, compared to the other emotions, participants made remarkably few attempts to understand or explain their intense disgust, one example of these situations was as follows:

*“Yes, just that, I just do not think that’s polite. That’s distasteful, disgusting. I do not need to see what you have in your mouth.” (Participant 2).*

#### 2.2.3 Secondary reactions

##### 2.2.3.1 Theme 6: safety behaviors

The concept of safety was generated in the transcripts, as participants described situations where they felt unsafe or engaged in behaviors that aligned with their personal sense of safety. It is important to distinguish this set of themes from the formerly discussed themes as these are not impulsive reactions but rather intentional safety behaviors. In the focus group discussions, “escape” involved physical distancing during a threatening event, while “avoidance” was a precautionary reaction to anticipated aversive situations. One of the most common examples was the use of ear plugs or headphones, as can be seen in the following example:

*“Yes, I have noticed that it is better to put on headphones or put in earplugs or whatever or go outside if possible.” (Participant 5).*

In addition, participants mentioned leaving rooms or creating distance between themselves and “the sources of the sound” during disturbing situations with trigger sounds. The tendency to avoid was mentioned in situations in which participants deliberately chose not to engage in specific events (i.e., not attending dinner parties, going to the movies etc.) or with certain people (i.e., people that they know who will likely trigger their misophonia). Furthermore, participants reported that they often try to mask the trigger sounds by either using headphones or chewing loudly themselves. The potential offensive contexts mentioned in the previous theme led participants to avoid or escape situations because of expected encounters with sounds and/or people. For instance, as a participant described, sleeping together with her/his partner was an offensive context, for which they had to make accommodations in their sleeping arrangements.

##### 2.2.3.2 Theme 7: fear

Two aspects of fear popped up from the current data set. First, being afraid of encountering trigger sounds and second, from one’s own possible (uncontrollable) reaction toward the sound or the person responsible for the sound (i.e., fear of consequences). The former mostly affected the daily lives of the misophonia sufferers and their families. The latter described a specific fear of not being able to control one’s own reaction and “going too far” in response to a sound or “sources of the sound.” An example of this fear can be seen in the following sentence from one of the participants:

*“But at such a moment, that person would be standing next to me, I could sometimes really do something to them and then I would be afraid of it afterwards because I am not like that and then I think: when will a moment come when I go too far, that I do something.” (Participant 5).*

Both types of fear reactions were anticipatory and influence the quality of life. Fear reactions were mainly reported when the participants mentioned an offensive context from which they cannot distance themselves. They described their experiences in offensive contexts with the phrases; “I was afraid that the person next to me might make the sounds,” “what would have I done if they made the sounds,” “I am afraid of my reactions,” “I know what will follow these sounds,” etc.

### 2.3 Discussion study 1

In order to better understand the excessive anger and disgust reactions in misophonia, the current study aimed to broadly explore meanings associated with trigger stimuli in misophonic individuals. Due to the explorative nature of the research question, we opted for a qualitative study consisting of two semi-structured focus group discussions with small groups of persons suffering from misophonia.

Although individual experiences of misophonia vary, the thematic analysis suggested key similarities in the meanings attached to trigger stimuli and subsequent reactions. Specifically, when trigger stimuli are produced by persons who are supposed to know how distressing the stimuli are to the misophonia sufferer (e.g., family members, partners, friends), misophonia sufferer experience to be *intruded on/violated* by the other person. Intrusion and violation have been described also in recently published case studies (e.g., Gregory and Foster, 2023; Gregory et al., 2024), suggesting that both meanings as derived in the present thematic analysis may be generalizable to most people suffering from misophonia. Our data showed that when experiencing “intrusion/violation,” the misophonia sufferer feels almost intentionally harmed. This finding corroborates earlier findings that misophonic anger is directed toward the other person, rather than to the sound itself (Edelstein et al., 2013), and with recent findings showing anger outbursts are associated with blaming others (so-called “externalizing appraisals,” see Wang et al., 2022). Similar sounds produced by animals and babies tend to trigger less emotion, likely because babies and animals are inherently innocent, or have no/less control over their actions and can therefore not be held responsible (Edelstein et al., 2013). Other findings suggest that persons with misophonia experience especially anger when trigger sounds are produced by people close to them (Bernstein et al., 2013; Dozier, 2015a). Interestingly, anger in response to a violation of borders in persons with misophonia is reminiscent of defensive rage that stems from the motivation to protect oneself and surroundings (Siegel and Victoroff, 2009). These observations led us to reflect on the theoretical distinctions between types of anger and identify the defensive nature of the emotional and behavioral responses expressed in our data. Different from predatory rage, defensive rage is characterized by a sudden increase in sympathetic activity and is highly impulsive (Siegel and Victoroff, 2009). In our focus groups, participants described emotional reactions that appeared to align more closely with defensive responses, as their anger was accompanied by a strong urge to escape the situation or shield themselves from the triggering sounds and associated individuals. These observations suggest that the extreme emotional reactions in misophonia may be driven by acquired, anger-provoking connotations (or: “meanings”) attached to the trigger sounds and the person producing them, especially when they are close to them. While the emotional experience described by participants is consistent with defensive rage, it is important to note that these emotions do not always manifest as overt aggressive behaviors. As reflected in statements such as “I flipped at that moment,” participants often describe an internal surge of anger or frustration, which may not necessarily escalate into physical or verbal aggression. This distinction between emotional response and actual behavior highlights the complex nature of misophonic reactions, where the intensity of the emotional experience can be intense, but not always outwardly expressed through aggression.

*Offense* and *disgust* showed up as major themes running through the focus group discussions, which converges with many other reports in the literature (Edelstein et al., 2013; Dozier, 2015a; Jager et al., 2020a; Siepsiak et al., 2020). Individuals with misophonia often perceive the actions of sound-producers as deliberate attacks against them, while also occasionally seeing these individuals, especially those less familiar, as only displaying poor manners (Taylor, 2017). This distinction underscores the theme of “offense,” as participants did not attribute intentionality to these actions, yet still considered the trigger sound producers as rude and lacking proper etiquette. Moreover, participants often attributed disgust not just to the sound itself, but primarily to the individuals producing those sounds with “poor manners,” whom they labelled as “dirty” or “disgusting.” This reaction was notably immediate and visceral, occurring without conscious mental processing. Participants frequently expressed intense disgust toward specific individuals, using phrases like “they disgust me” or “this person disgusts me.” In comparison to other emotions, focus group participants did not try to elaborate or to reason their feelings of disgust, which is in line with the unreasoning disgust hypothesis of Russell and Giner-Sorolla (2011). The hypothesis states that compared to (moral) anger, (moral) disgust is less likely to be justified with cognitively elaborated reasons (Russell and Giner-Sorolla, 2011). Therefore, even though sometimes the person with misophonia felt uncomfortable with their own reactions and find them excessive, they still labelled the actions of the person producing the sounds as being impolite, disgusting or unintentionally offensive (Taylor, 2017; Brout et al., 2018). Furthermore, this complex reaction to trigger sounds, characterized by immediate disgust toward the individual producing the sound, raises questions about the nature of disgust in misophonia. While responses seem largely visceral, it remains unclear whether this disgust is moral or sensory. Some research suggests it may be a moral disgust (Iskander et al., 2023; Norena, 2024), but further investigation is needed to clarify whether the disgust in misophonia aligns more with moral or visceral disgust.

The *lack of autonomy* was revealed as the last meaning, referring to having control over one’s own emotions and behavior, over the (offensive) behaviors of others, and even over one’s life. Reports in the literature indicate early adolescence as the most typical age of onset for misophonia (Johnson et al., 2013; Dozier, 2015a; Kumar et al., 2017; Rouw and Erfanian, 2018; Guzick et al., 2023). At this age, children may experience an increasing and often unmet need for autonomy and independence from their parents (Young et al., 2019; Hu et al., 2021). At the same time, self-control and emotion regulatory capacity are still underdeveloped (Young et al., 2019; Warschburger et al., 2023). Both may set the stage for strong learning experiences through which anger and disgust reactions can become associated with misophonic triggers. In addition, a possible motivational conflict can be observed in the scenarios in which a person requires a certain level of autonomy, yet is still bound by their parents. Potentially, the underlying motivation to gain autonomy may have been present before the development of misophonia.

Apart from “intrusion/violation of borders,” “anger/defensive rage,” “offense,” “disgust,” and “lack of autonomy,” major themes such as *(anticipatory) fear*, *escape* and *avoidance* also constructed. During the final stage of the thematic analysis discussions, we consolidated the themes of escape and avoidance behaviors into a single overarching theme named “safety behaviors.” Consistent with the literature (Edelstein et al., 2013; Cavanna and Seri, 2015; Palumbo et al., 2018; Dibb and Golding, 2022), fear did not show up as an instant primary emotional reaction to trigger stimuli in a similar way as “anger/defensive rage” and “disgus” did. Instead, fear was referred to more in terms of concerns of what could happen if persons would be trapped in a context in which trigger stimuli might occur, how to avoid or escape from such contexts, and the potential social consequences if one would be unable to control aggressive impulses. Thus, in contrast to other sound-related disorders such as hyperacusis or bothersome tinnitus, our findings suggest that fear seems less of a central, primary emotion in response to the misophonia trigger sounds.

The semi-structured focus groups were directed specifically at understanding misophonic events exploring meanings of the trigger stimuli leading to excessive and instantaneous emotional reactions. Longer term consequences of repeated misophonic experiences in the participants’ interpersonal, professional, and emotional functioning, were not extensively discussed and were not represented in the findings from the conducted thematic analysis. That does not make them unimportant, however. Feelings of guilt, shame, helplessness/sadness were occasionally mentioned in the focus group discussions and may importantly relate to the overall impact of misophonia on the sufferers and their families. This aligns with prior literature reporting “internalizing appraisals” (Gregory and Foster, 2023), which are also important to recognize as an important aspect of suffering in persons with misophonia, other than the instant emotional reactions (anger, disgust) and meanings in response to triggers that were the focus of this study.

Qualitative designs offer flexibility to explore various aspects of a topic, but come with a number of limitations that need to be acknowledged. First, the data collected during the focus groups are based on open-ended questions, and participants have control over the information they share. During the discussions, participants may occasionally veer off-topic and may influence each other with their answers. Therefore, answers may have been different in an individually conducted interview. Participants may have been inclined to provide socially desirable or conformist answers. For instance, because participants were invited to think back to a situation where they felt bad because of the sound, there is a chance that thoughts and behaviors elicited by stronger emotions might be overrepresented. Even though we made concerted efforts to minimize these limitations by having the moderator adhere to the script as closely as possible, future research can rely on diverse qualitative designs (i.e., focus groups, interviews, surveys etc.) to tackle these issues. Second, although the researchers were engaged to maintain objectivity and made decisions solely based on the data during the thematic analysis, it is worth acknowledging that alternate themes, slightly different labels for existing themes, and slightly different relationship between themes may have resulted from a thematic analysis when performed by other researchers.

In line with Braun and Clarke’s guidelines on thematic analysis (Braun and Clarke, 2006; Braun and Clarke, 2019), we conducted a reflexive thematic analysis aimed at exploring the potential meanings and emotions associated with misophonia trigger sounds. While our analysis was not driven by pre-existing themes, it was guided by our theoretical interest in understanding meaning formation processes, which aligns with a deductive approach. The analysis was interpretative rather than descriptive, meaning that we generated themes by actively reflecting on and analyzing the data with an overarching goal of creating meaning themes. Since completing our analysis, Braun and Clarke (2022) have highlighted the distinction between “topic summary” and “meaning-based interpretive story” themes, with an emphasis on choosing between these approaches. Although this updated guidance was not available at the time of our analysis, our thematic approach aligns closely with their concept of “meaning-based interpretive story” themes, as we prioritized deeper interpretative insights over descriptive summaries.

Last but not least, the focus groups consisted of a small number of individuals. The low number of participants facilitated a safe environment for the participants and ensured the chance for everyone to have open discussions. However, while this approach provided valuable insights, it remains imperative to verify the generalizability of the findings through a confirmatory study encompassing a broader population of individuals exhibiting misophonic symptoms across different severity levels. To address this need, we conducted a larger scale questionnaire study, including main the meanings generated in this study, to focus on the generalizability of the findings.

## 3 Study 2

The primary objective of study 2 was to validate the findings from our exploratory qualitative study (study 1) using a large, independent sample. Specifically, we aimed to (1) assess the extent to which the meanings of “intrusion/violation,” “offense,” and “lack of autonomy” are experienced in response to prototypical misophonic triggers, and (2) determine whether these experiences correlate positively with the severity of misophonic symptoms. Additionally, the study aimed to explore the relationships among these meanings, primary reactions, and secondary reactions (i.e., the themes identified in the thematic analysis of Study 1) and to uncover a possible meaningful latent structure through factor analysis.

### 3.1 Methods and materials

#### 3.1.1 Participants

A total of 463 participants initially completed 2 questionnaires; after accounting for dropouts and excluding responses completed in less than half the median time to ensure data integrity, the final sample consisted of 431 participants (359 female) with an average age of 18.42 years (range: 17–35; 86% Belgian, 14% international students). Notably, participants’ misophonia status was not determined beforehand, meaning they were included without prior knowledge of whether they did or did not have misophonia. All participants were Dutch-speaking first year Bachelor of Psychology students. Participation was incentivized by offering course credits. Informed consent was obtained from all participants, with additional parental consent required for those aged 17. The study received ethical approval from KU Leuven University, ensuring adherence to ethical research standards [SMEC; approval number is G-2023-7044-R5 (MIN)].

#### 3.1.2 Procedure

Participants answered a range of questions using the Qualtrics online platform. First, they rated a list of 19 custom-made items that aimed to assess participants’ prototypical experience (meanings, reactions) when being exposed to misophonic triggers. Participants received the following textual instructions to rate their prototypical experience for each item “*Please indicate to what extent you have the following experiences when hearing sounds that other people make when they breathe, eat (chewing, slurping, smacking, swallowing, etc.), clear their throat, cough, cut their nails, sniff (pick up their nose), or when they repeatedly click a ballpoint pen in and out, tapping their fingers on the table. When hearing these sounds….”* Upon reading this text, participants were shown each item to be rated on a horizontal 0–100 visual analog scale, with the following labels: 0 (“not at all”), 50 (“to a certain extent”), and 100 (“very much”) (see Table 3 for each item). The 19 items consisted of 6 items reflecting meanings as identified in the thematic analysis, with 2 items referring to the “intrusion/violation” theme (“intrusive,” “violated”), 1 item referring to the “offense” theme (“offended”) and 3 items referring to the autonomy theme (“lack of autonomy,” “feeling trapped,” “lack of control”). The primary (“anger,” “defensive rage,” “disgust”) and secondary (“afraid,” “desire to avoid,” “need to escape”) reactions were captured in 6 additional items. The 9 remaining items were added as explorative or slightly contrasting items, i.e., emotions that were not generated during the thematic analysis of study 1 (“relaxed,” “guilty,” “enthusiastic,” “calm,” “sad,” “happy and excited,” and “peaceful”).

**Table 3:** Themes (meanings, reactions) identified in Study 1 translated into Items in Study 2 with their corresponding categories, means, standard deviations, medians, range and correlation with A-Miso-S scores across the whole sample.

Themes ^a^	Item category	Items as presented to participants^b^	Mean rating	SD	Range	Median	A-Miso-S score correlation^c^
Intrusion/Violation	Associated Meaning	I experience the sounds as intrusive	53.32	27.54	0–100	56	0.46
		I experience being violated	16.23	22.04	0–100	5	0.45
Offense	Associated Meaning	I feel offended	13.74	19.44	0–100	5	0.29
Lack of Autonomy	Associated Meaning	I experience a lack of autonomy	17.38	22.61	0–100	6	0.42
		I experience a lack of control	35.28	30.95	0–100	28	0.46
		I feel trapped in the situation	40.81	30.62	0–100	40	0.47
Anger/Defensive rage	Primary Reaction	I feel anger	44.32	30.05	0–100	45	0.52
		I experience a defensive anger (rage)	29.03	29.1	0–100	20	0.51
Disgust	Primary Reaction	I feel disgust	43.71	30.73	0–100	49	0.27
Safety Behaviors	Secondary Reaction	I want to avoid the sounds	70.03	26.93	0–100	76	0.4
		I experience the need to escape	49.48	30.11	0–100	50	0.4
Fear	Secondary Reaction	I feel afraid	7.26	15.22	0–100	1	0.29
	Exploratory	I feel calm	19.12	21.39	0–100	11	−0.33
		I feel relaxed	18.87	19.52	0–87	10	−0.37
		I feel peaceful	11.67	16.65	0–94	4	−0.21
		I feel sad	10.31	17.44	0–100	2	0.44
		I feel guilty	9.27	18.44	0–100	2	0.32
		I feel happy and excited	8.73	14.06	0–85	2	−0.16
		I feel enthusiastic	7.32	12.15	0–56	1	−0.17

a Themes identified in Study 1.

b Themes converted into scorable items in Study 2.

c All correlations between items and A-Miso-S scores were significant at *p* < 0.001.

Next, participants completed the A-Miso-S (Schröder et al., 2013) to assess the presence and severity of their misophonia symptoms (see method section of study 1 for a description of the A-Miso-S).

#### 3.1.3 Statistical analysis

To examine the association between the items (meanings and reactions identified in study 1) and misophonia symptom severity, Pearson correlations were calculated between A-Miso-S scores and each of the meanings, primary and secondary reactions, and explorative items. To investigate how the rated meanings uniquely related to A-Miso-S scores, a multiple regression analysis was conducted with participants’ answers on the 6 meaning items as predictors and A-Miso-S scores as the dependent variable. Next, a factor analysis was conducted to investigate whether the meanings and primary and secondary reactions could be meaningfully grouped into one or more underlying (latent) factors. Factors with eigenvalues >1 were retained and an oblique rotation (quartimin) was applied.

### 3.2 Results

#### 3.2.1 Descriptive statistics

Out of 431 participants, 39.7% (171) scored between 0 and 4 (subclinical symptoms), 45.2% (195) scored between 5 and 9 (mild symptoms), 11.6% (50) scored between 10 and 14 (moderate symptoms), 2.8% (12) scored between 15 and 19 (severe symptoms), and 0.7% (3) scored between 20 and 24 (extreme symptoms) on the A-Miso-S questionnaire. The four meaning/reaction items rated highest were: “desire to avoid the sounds,” “intrusion,” “need to escape,” and “anger,” see Table 3 for the descriptive statistics of all items.

#### 3.2.2 Associations of meaning items with meanings and A-Miso-S scores

**Figure 1:**
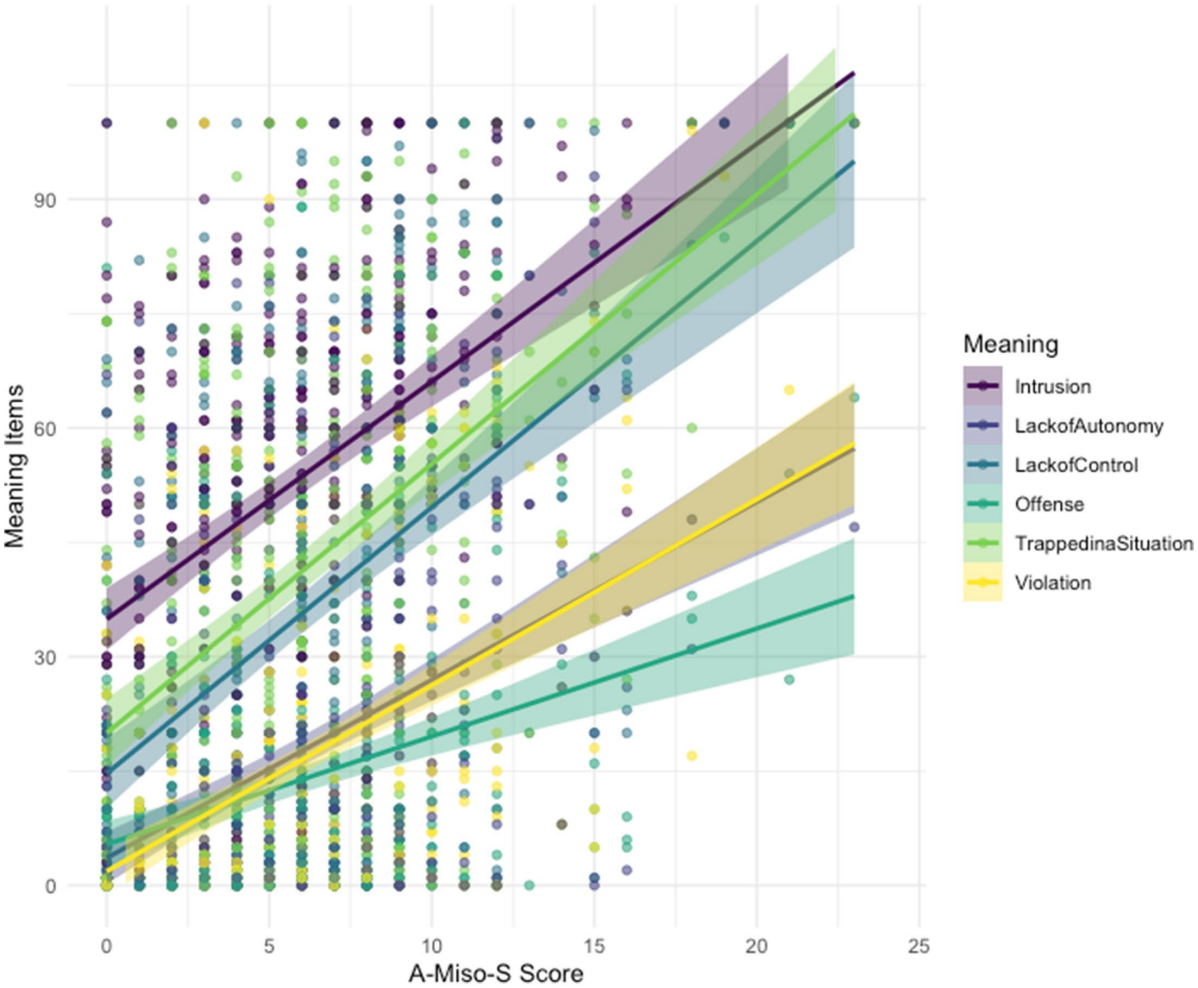
Correlations between A-Miso-S scores and the selected rating items (meanings).

The rated meaning items showed significant, moderately positive associations with A-Miso-S scores (see Figure 1; Table 3) and with each other (see Supplementary Tables S1, S2).

A multiple regression analysis was conducted to examine how the rated meanings (intrusion, violation, lack of autonomy, feeling trapped in a situation, offense, lack of control) as predictors were uniquely related to A-Miso-S scores as the dependent variable. The model demonstrated a significant association between the predictors and A-Miso-S scores [*F*(6, 424) = 38.3, *p* < 0.001], explaining 35.15% of the variance in A-Miso-S scores. Specifically, intrusion (*β* = 0.027, *p* < 0.001), lack of control (*β* = 0.021, *p* = 0.002), violation (*β* = 0.026, *p* = 0.007), feeling trapped in a situation (*β* = 0.019, *p* = 0.007) and lack of autonomy (*β* = 0.019, *p* = 0.033) were significant predictors of A-Miso-S scores. However, offense did not significantly predict A-Miso-S scores (*β* = 0.011, *p* = 0.237). The model’s intercept was found to be significantly different from zero (*β* = 2.013, *p* < 0.001).

**Figure 2:**
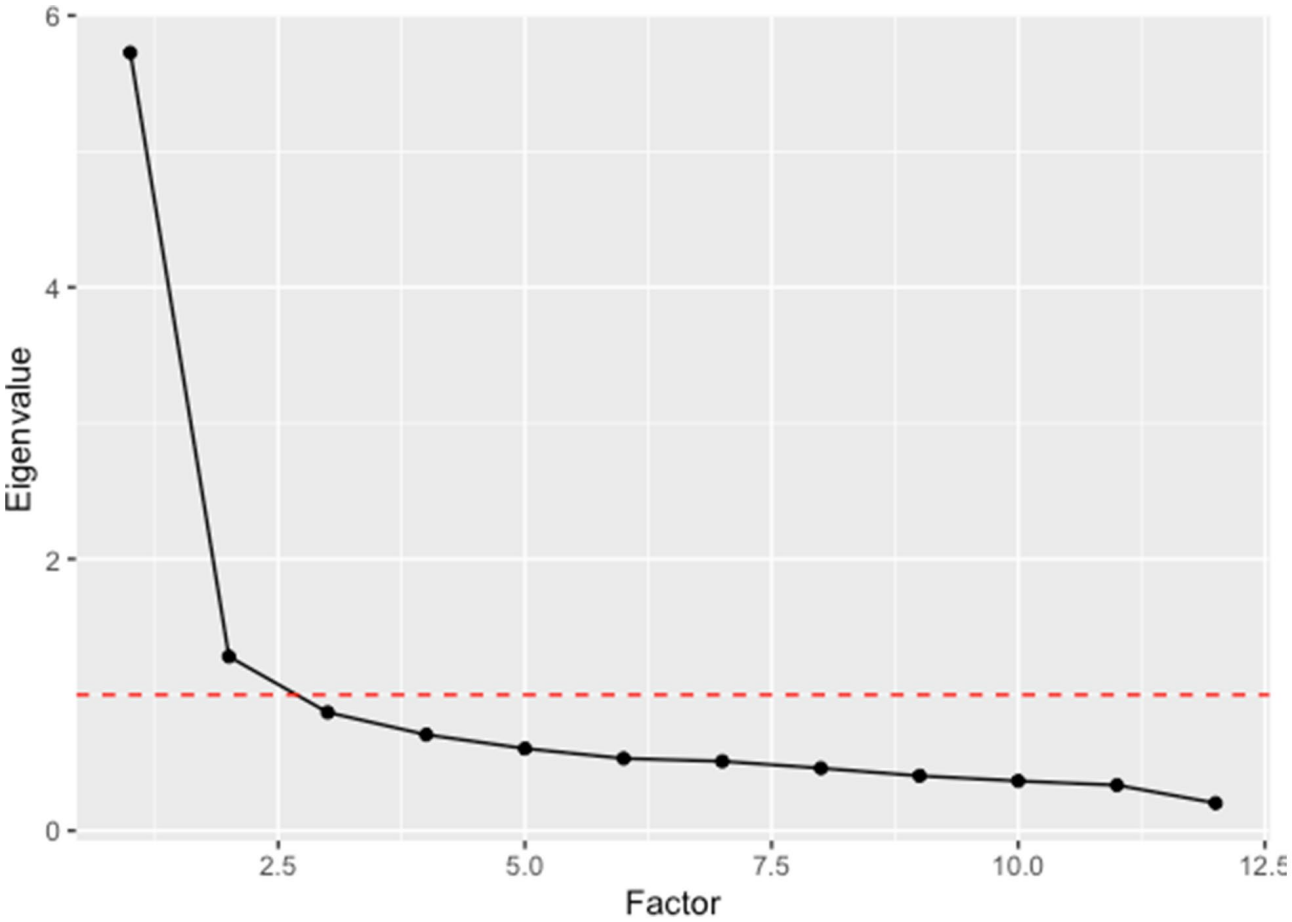
Scree plot of eigenvalues.

The dataset’s suitability for factor analysis was confirmed by the Kaiser-Meyer-Olkin (KMO) measure (MSA = 0.92) and Bartlett’s Test of Sphericity (*x*^2^ = 4547.29, *p* < 0.001), indicating significant common variance among items referring to meanings and (primary/secondary) reactions. Individual item MSAs ranged from 0.84 to 0.97, surpassing the 0.5 threshold. The scree plot identified two factors with eigenvalues >1 (see Figure 2). The eigen values for Factor 1 and Factor 2 were 5.73 and 1.28, respectively.

An exploratory factor analysis was conducted using maximum likelihood estimation with oblique quartimin rotation. For the first factor, the three items with the highest factor loadings were “desire to avoid the sounds” (0.91), “intrusion” (0.61), and “disgust” (0.61). These loadings were also unique to factor 1, which we therefore will label “Avoidance of intrusive/disgusting sounds.” The second factor showed unique and high loadings on “lack of autonomy” (0.74), “violation” (0.72), and “offense” (0.6). This second factor will further be called “Autonomy/Violation.” Table 4 displays the complete list of the factor loadings on all items.

**Table 4:** Factor loadings for exploratory factor analysis with the meanings from study 1 (*N* = 431).

Item Name	Factor	
	1	2
Intrusion	0.61	0.2
Violation	0.08	0.72
Offense	−0.07	0.60
Lack of autonomy	−0.04	0.74
Lack of control	0.35	0.38
Trapped in a situation	0.54	0.24
Anger	0.51	0.42
Defensive anger (rage)	0.34	0.51
Disgust	0.61	0.05
Desire to avoid the sounds	0.91	−0.16
Need to escape	0.55	0.19
Afraid	−0.08	0.56
% of variance	0.26	0.24

Maximum likelihood extraction method was used with an oblique quartimin rotation. Factor loadings of > 0.50 are bolded.

Together, the two factors explained 50% of the cumulative variance, with Factor 1 explaining 26% and Factor 2 explaining 24% of the total variance.

### 3.3 Discussion study 2

In study 2, we aimed to confirm our qualitative results using a quantitative approach. The themes (meanings, primary and secondary reactions) identified via the thematic analysis in study 1 were converted into scorable items, allowing to quantitatively explore the prevalence of the uncovered meanings and their association with misophonia symptoms severity in a larger, independent sample. We also explored whether factor analysis on the rated meanings and reactions would show an underlying, meaningful latent factor structure.

The responses of the 431 participants to the A-Miso-S questionnaire demonstrated that there was a considerable percentage (15.1%) of people scoring above the threshold of 10 in this unselected sample of young adults. This is in line with other studies investigating the occurrence of misophonia in students and general populations (e.g., Wu et al., 2014; Patel et al., 2023; Vitoratou et al., 2023).

The descriptive and correlational findings from study 2 generally confirm that in this large sample of young adults, the constructed meanings and reactions to misophonic stimuli as identified in study 1 are indeed prevalent. The meaning items “intrusion” and “feeling trapped in a situation,” received the highest ratings, with median scores of 56 and 50 on a 0–100 scale, respectively. These ratings are surprisingly high for our sample, in which 84.9% of the participants scored below the cut-off of 10 for moderate misophonia symptoms on the A-Miso-S. Apparently, also persons with lower A-Miso-S scores experiencing to some extent “intrusion” and “feeling trapped in a situation” upon exposure to misophonic stimuli, though to a lesser degree than persons with more severe misophonia symptoms (see Figure 1 and Table 3). In contrast, the meaning items “lack of control,” “violation,” “loss of autonomy,” and “offense” were scored generally lower and appear more unique to persons with more severe misophonia symptoms (see Figure 1). With the exception of “offense,” all items referring to meanings showed a similar, significant moderately positive association with A-Miso-S scores and each contributed independently to the prediction of misophonia symptoms severity. This corroborates the idea that meanings linked to misophonic trigger stimuli are an inherent part of the misophonic experience. The observation that the “offense” item showed a weaker correlation with misophonia symptoms and was not found to be an independent predictor of misophonia symptom severity in the multiple regression, suggests that the study-1 theme “offense” as being a separate meaning-theme, may reflect a peculiarity of the persons in the focus groups that is not generalizable to others. In sum, the meaning themes of “intrusion/violation” and “lack of autonomy” seem clearly reproduced as distinct meanings that relate to misophonia symptoms in the large, independent sample of study 2.

Apart from this, findings of study 2 also support “anger” and “disgust” as primary reactions to misophonic stimuli, which is in line with other reports in literature (e.g., Edelstein et al., 2013; Cavanna and Seri, 2015; Schröder et al., 2017; Palumbo et al., 2018; Dibb and Golding, 2022). Although “disgust” had an overall higher median rating than “anger,” it showed a weaker correlation with misophonia symptoms severity (see Table 1). This pattern of findings suggests that misophonic stimuli do trigger disgust also in persons who score low on misophonia symptoms, while anger seem more exclusively characteristic for persons suffering from more severe misophonia symptoms. While disgust may play a role in misophonia, our results imply that it may not be as central as anger in characterizing the disorder.

As study 2 assessed the immediate (primary) reaction of participants to misophonic stimuli, the present data do not allow direct confirmation of our study 1’s finding that “fear” and “safety behaviors” are secondary reactions. Nonetheless, the low ratings of the fear item “afraid” confirms the finding of study 1 and other findings in the literature that fear is indeed not a primary reaction to misophonic sounds akin to phonophobia or hyperacusis (Schröder et al., 2013). Surprisingly, the item “desire to avoid sounds” had the highest median rating of all rated items (76 on a 0–100 scale), which suggests that an avoidance tendency is also a primary reaction to misophonic sounds that may have been missed in the thematic analysis on the data of study 1. Whether “avoidance” can also be a secondary, less impulsive reaction, cannot be discerned from the data of study 2, as we did not assess secondary reactions. Important to note here is that the A-MISO-S has an item on avoidance as well, therefore, the high correlation of “desire to avoid sounds” and A-MISO-S scores is likely partially due to content overlap. Such content overlap was not present for the other rated items.

In addition to the items identified in the thematic analyses, we included both negative and positive exploratory items in our questionnaire, and they correlated in the expected directions. For example, the negative emotions “sad” and “guilty” were positively correlated with misophonia severity, while positive emotions such as “happy and excited,” “enthusiastic,” “peaceful,” “relaxed,” and “calm” were inversely correlated. These findings for the negative emotions align with previous research indicating that misophonia sufferers also experience sadness and guilt because of their anger outbursts (Wang et al., 2022; Dibb and Golding, 2022).

Factor analysis on the items referring to meanings, primary and secondary reactions further underscored the salience of themes identified in the initial qualitative study and how they relate to the reactions. Two latent factors together explaining 50% of the variance in the data were retained. The first factor encompassed the meaning item “intrusion” and had also high loadings on “desire to avoid sounds,” “need to escape,” “feeling trapped,” “disgust,” and “anger” prompting the designation of this factor as “Avoidance of intrusive/disgusting sounds.” Consistent with previous literature, this factor confirms that when trigger sounds are experienced as intrusive, persons experience disgust and anger and are strongly and instantaneously motivated to avoid them (Edelstein et al., 2013; Schröder et al., 2017; Jager et al., 2020a; Rouw and Erfanian, 2018). For the second factor “Autonomy/Violation,” the meaning items “lack of autonomy,” “violation,” and “offense” had the highest factor loadings, followed by the emotional reaction items “fear” and “anger/defensive rage.” Whereas the meanings of “intrusion” and “violation” were collapsed in one overarching theme based on the thematic analysis of study 1, findings from the factor analysis suggest they rather reflect separate meanings that are associated with a different pattern of reactions. Specifically, “intrusion” relates uniquely to “disgust” and “avoidance” and seems also prevalent to some degree in persons with no or mild misophonia symptoms. “Violation,” on the other hand, coincides with more extreme forms of “anger” and is present only in those with more severe misophonia symptoms.

While our study offers valuable insights into the relationships between the emotions and meanings attached to the trigger sounds of misophonia, there are several limitations to consider. Our sample was primarily composed of Bachelor students, with a large proportion being female, which may affect the generalizability of the findings to a more diverse population. Given that theory of mind and perspective-taking abilities continue to evolve into late adolescence, suggesting ongoing development in understanding others’ thoughts and intentions (Dumontheil et al., 2010), the age of our sample (primarily 18-year-olds) may influence social perspective-taking and blame attribution, potentially impacting the emotional constructs examined in our study and limiting the generalizability of our findings to older or younger populations. Therefore, more research investigating the onset and developmental trajectory of misophonia, is needed to better understand the interplay between early symptom emergence and the cognitive appraisal mechanisms that contribute to meaning formation in misophonic reactions. Of course, our research is inherently limited without observing the entire developmental trajectory of misophonia, highlighting the importance of longitudinal studies to fully capture these processes.

Additionally, terms derived from the themes’ description (thematic analysis study 1) were directly incorporated into custom-made items. Despite our efforts to ensure clarity of the items, encapsulating these items into concise terms was challenging. Particularly noteworthy in this respect is the item “offense.” In the focus groups, the “offense” theme primarily described being offended by the sound or environments where the trigger sounds might be present rather than perceiving disrespect from an intentional source of the sound. Moreover, the theme of “offense” encompasses misophonia experiences such as distress from unintentional triggers, perceiving them as offensive, and feeling offended in environments considered normal and safe by others (e.g., restaurants, cinemas, home). The item in our questionnaire might have insufficiently captured all this. Furthermore, another potential limitation regarding the custom-made items is that “loss of control” may overlap conceptually with the A-Miso-S item regarding “control over thoughts about misophonic sounds.” However, it is important to note the distinction that while the A-Miso-S item focuses on obsessive, intrusive thoughts in the absence of sounds, our item captures a more immediate loss of control over actions in response to misophonic triggers. We also conducted a sensitivity analysis by repeating the factor analysis without including “loss of control” as a variable. The results remained largely consistent, suggesting that the factor structure was stable even when this item was excluded.

In summary, findings from study 2 confirm that “intrusion,” “violation,” “offense,” and “lack of autonomy” are core meanings attached to prototypical misophonic triggers and characterize the misophonic experience. “Disgust,” “anger,” and a strong “avoidance” tendency show up as primary reactions to trigger sounds that are experienced to be intrusive, whereas more extreme forms of anger (“defensive rage”) are experienced when trigger-sounds signify “violation,” “offense,” and/or a “threat to one’s autonomy.”

## 4 General discussion

Although misophonia is increasingly gaining attention from both the general public, clinicians and researchers, much remains to be uncovered (Cowan et al., 2021; Gregory and Foster, 2023; Gregory et al., 2024; Norena, 2024; Rosenthal et al., 2023). The present studies aimed to shed light on why anger and disgust are the core primary reactions to misophonic triggers. We found that misophonic trigger stimuli convey strong and specific meanings to persons suffering from misophonia, namely: “intrusion,” “violation,” “offense,” and “lack of autonomy.” These meanings seem an integral part of a cohesive and debilitating emotional representation of misophonic experiences and seem inherently linked to the extreme anger and disgust reactions that characterize misophonia.

The presently identified meanings may inspire further work on etiological mechanisms of misophonia. Specifically, Pavlovian conditioning has been proposed repeatedly as an etiological mechanism of misophonia (e.g., Jastreboff and Jastreboff, 2002; Palumbo et al., 2018), but this idea has remained unsupported by empirical evidence. Following the conditioning account, a trigger stimulus would have become a conditioned stimulus (CS) evoking anger and disgust as a conditioned reaction (CR) via Pavlovian conditioning (Jastreboff and Jastreboff, 2002; Dozier, 2015a; Palumbo et al., 2018). Until now, the literature has remained surprisingly silent on what type of unconditional stimulus (US) could have been associated with the trigger stimulus (CS) in the initial learning experience to elicit anger and disgust (see also Cowan et al., 2021). Because of this unclarity in the US conceptualization, there is at present no laboratory model that can put the conditioning account of misophonia to the test. The present findings may offer an avenue to tackle this problem by hypothesizing that trigger sounds may have acquired the core meanings of “intrusion,” “violation,” “offense” and/or “lack autonomy” via Pavlovian learning, thereby explaining why anger and disgust (rather than fear) constitute the core emotional reaction toward them (Edelstein et al., 2013; Bernstein et al., 2013; Dozier, 2015a; Muller et al., 2018; Potgieter et al., 2019; Ferrer-Torres and Giménez-Llort, 2022). The concept of referential learning, a particular type of Pavlovian conditioning, might provide insight on how meanings can get associated to stimuli. In referential learning, meaning aspects of the US (e.g., a parent repulsing his/her child’s need for autonomy by forcing the child to stay at the dinner table) are transferred to the CS (smacking sound of the parent at the dinner table) (Baeyens and de Houwer, 1995; Baeyens et al., 2001; Ludvik et al., 2015). The best-known instance of referential learning is the learning of likes/dislikes, also named evaluative conditioning. In such learning, individuals for example learn to dislike a certain food after a food poisoning experience. After this type of learning, people do not expect to get food poisoning again when consuming similar food (there is no “predictive” or “signal” learning), they simply acquired a dislike of this food-type and find it even disgusting. Much like misophonia in fact, where persons do not report a particular expectation of an aversive event (US) to happen upon perceiving a trigger stimulus (CS). Instead, trigger stimuli are inherently experienced as intrusive, offensive, violating one’s borders, and give possibly therefore rise to instant (impulsive) anger and disgust. We propose *referential learning* as an interesting avenue to further explore etiological mechanisms of misophonia. Future research might include the development a laboratory paradigm to model in human participants the transfer of meanings identified in the present study (intrusion, violation, offense, lack of autonomy) onto prototypical misophonic trigger stimuli.

Based on our findings that misophonia severity is related to trigger stimuli conveying particular meanings to the misophonia sufferer, changing those meanings seems a promising and logical psychological treatment avenue. A technique that is theoretically apt to break down meanings attached to trigger stimuli is counter conditioning (Kerkhof et al., 2011), a procedure in which an opposing US is paired with the CS. This technique has been proposed and applied already in the context of misophonia (e.g., Dozier, 2015b; Jager et al., 2020b; Mattson et al., 2023), but not with the specific goal to break down the disabling meanings of intrusion, violation, offense, and lack of autonomy attached to trigger stimuli. A fictitious application of such counter conditioning could be creating a pleasant context (e.g., dinner party) where the misophonic individual experiences autonomy (prepared the food him- or herself, selected and invited guests, chose the music, walking dinner party with buffet) and attaches another meaning to the party guests’ eating sounds (e.g., “my friends really adore the food I prepared all by myself!”), thereby offering a strong learning opportunity that weakens the disabling meaning representation around, in this case, eating sounds. Psychological treatment strategies for misophonia seem predominantly directed at regulating emotions and distress, rather than at changing what trigger stimuli mean to the misophonia sufferer. As an example, mindfulness-based therapies and acceptance and commitment therapy (ACT) for misophonia aim to help patients tolerate distress and detach from misophonic reactions (Cecilione et al., 2021). Another proposed treatment, the Unified Protocol, targets both emotional regulation and cognitive flexibility, addressing the complex emotions triggered by sounds in misophonia (Rosenthal et al., 2023). Also, CBT protocols for misophonia are mainly directed at improving emotional regulation through a variety of techniques, including relaxation/arousal reduction, attentional training, cognitive restructuring and stimulus manipulation (Jager et al., 2020b; Mattson et al., 2023). Interestingly, some authors report mere exposure to trigger stimuli, as one would apply in fear-based pathology, to be a less effective treatment strategy for misophonia (Cecilione et al., 2021; Schröder et al., 2017; but see Frank and McKay, 2019), and to occasionally even increase misophonia symptoms (Schröder et al., 2017). Whereas mere exposure can effectively disconfirm fearful expectations (e.g., strong expectation that a dog will attack, it is less likely to change the triggers’ associated meanings of violation, intrusion, lack of autonomy, and offense). Indeed, these US-like meanings may present themselves with each exposure to trigger stimuli spontaneously, with the inherent risk of strengthening the disabling emotional representation. Counter conditioning of specific meanings conveyed by trigger stimuli may therefore constitute a better alternative treatment strategy that also nicely complements strategies aimed at improving emotional regulation.

In conclusion, the presently identified meanings of violation, intrusion, offense and autonomy that misophonia sufferers attach to their triggers, help understanding why extreme anger and disgust constitute the core emotional reactions characterizing misophonia. The identification of these meanings offers opportunities to further fertilize a theoretical learning account of etiological mechanisms of misophonia, and pave the way toward more mechanism-informed treatment strategies.

## Data Availability

The raw data supporting the conclusions of this article will be made available by the authors, without undue reservation.
