# Supplementary material for: Understanding the misophonic experience: a mixed method study
Source: Front Psychol. 2025 Feb 5;16:1493676. doi: 10.3389/fpsyg.2025.1493676 (PMC11835947; doi:10.3389/fpsyg.2025.1493676)
Supplement: Supplementary file 1 [file Table_1.DOCX]

| **MISOPHONIA PROTOCOL**  **EXPERIENCE EXPERTS** | | | | | | |
| --- | --- | --- | --- | --- | --- | --- |
| **STEP** | **Test Phase** | |  |  |  | **Time**  **(min)** |
| 0 | **ONLINE PRETEST PHASE (± 1 week before the test)** | | | | | **30** |
| 0a | After a person registers via email:   1. Assign them a participant ID 2. Add them to the encrypted participant file (add name of the file)   Send them an email with the following information: assigned participant ID, doodle link for arranging the meeting, link to Qualtrics (insert link here when available), and the informed consent form | | | | |  |
| 0b | If they will be included in the focus groups:   1. Send them an email with the following information: link to the meeting, details of date and time of the meeting, details on how to log-in and use Microsoft Teams 2. Add them to the participant file of the related group 3. Make sure you have the signed and scanned informed consent form   If they will be excluded based on availability:   1. Thank them for their time and let them know the first group is full, ask them if you can contact them later for other studies 2. Save their contact information on the encrypted participant file with the results of the Qualtrics questionnaire 3. Save their informed consent form   If they will be excluded based on the questionnaire outcomes:   1. Thank them for their time and let them know the groups are full 2. Delete them from the encrypted participant file   Delete their informed consent form | | | | |  |
| 1,2,3,4 | **ONLINE FOCUS GROUP PHASE** | | | | | **±180** |
| 1a | **Instructions**  *Good morning/afternoon everyone, first of all, thank you so much for responding to our invitation and taking the time to fill out questionnaires and allow us to talk to you today. I am (name of the moderator), today’s focus group moderator. We also have here Moderator 2, Observer 1 and Observer 2 who are joining the session as the researchers. (Name of the 2^nd^ moderator) will be your second contact person, if you have any personal questions or connection related problems you can write to them on the private chat.*  *So let me tell you what we are going to do today. This day will consist of three parts and it will take approximately 2.5 hours. We aim to find out more about how you experience misophonia and talk about the emotions you go through. I also want to let you know that; these discussions will not be shared anywhere other than for analysis reasons. After we record these sessions, we will anonymize your responses. I also want to stress that you are here voluntarily and you have the right to leave at any given moment if you feel uncomfortable.*  *Firstly, we kindly ask you to keep your camera on during the whole session. During some parts you might want to turn the camera off, but we will let you know when that might be helpful.*  *Your microphones are turned off automatically, as you can see/notice. This is so that we will be able to hear to everyone who wants to join. If you want to add to the conversation, please feel free to raise your hand. To create a safe space for everyone, it is also important to remember to not interrupt each other while speaking and respectful to each other’s opinions and feelings. If you are having trouble with the technical use of zoom please let us know in the chat! to the second moderator (Name of the 2^nd^ Moderator). And as you have read in the informed consent forms, we will be recording the session in order to be able to analyze later. So, if everyone is ready, I will start the recording now.*  **START RECORDING**  *Maybe before we start, we can do a little round of names and two sentences about ourselves. I can start, my name is (Name of the Moderator) and I work (do my masters) in the health psychology department of KU Leuven. On the top corner of my screen, there is Moderator 2 / Observer 1/ Observer 2. And we can maybe continue with (name of a participant)….*  **For the participants say their name for them to speak**  *Since we got to know our names, now I can give you a little introduction to our study. Current study aims to explore the mechanisms of misophonia / hyperacusis / bothersome tinnitus. Through this focus group we aim to find out possible reasons why certain sounds are more disturbing for some people than for others. More specifically, the underlying emotions, thoughts and meanings might be responsible for aversive reactions to harmless everyday sounds. We are interested in what the sounds mean to you as you are experiencing them, or what it is about the sound that is specifically causing you to respond in these ways. During this discussion session, we will ask you a series of questions, for some of them we will ask you to use the poll options, and for some you will discuss the questions in smaller groups. If everyone is ready, I would like to start with the first part of the discussion. Are there any questions right now…?* | | | | | **15** |
| **1^ST^ SET OF QUESTIONS: THINKING ABOUT EVENTS** | | | | | | |
| 1b | **AIM**  We will ask them to imagine the scenario again in order to remember the thoughts and feelings. | **STAGE 1 THINKING ABOUT THE EVENT**  *I would like to ask each of you to think back of a situation in which you felt bad because of the sound. It can be a recent or a remote memory, both are fine, as long as you can remember it vividly, because we will be asking questions about what you experienced back then later on.*  *So, try to think of a situation that you can remember vividly. Take your time to come up with such situation. If you prefer, you can turn of the camera for a while, maybe that makes it easier to bring the situation back in your mind. If you like, you can close your eyes for a while. If you have such situation, I’d like to ask you to imagine yourself for a while you are back there, and try to experience and feel what you actually felt, this will help you to remember the situation better and to answer the questions we will ask later on. We will take a few minutes so that you can think of and select a situation that you vividly remember. I will ask in about 3-5 minutes to turn on the camera again, we will then check whether you have found such situation. Thus, take your time to get a situation back into your mind, try to remember and feel how it was. ….* | | | | **2** |
|  |  | **A thinking break for 3 – 5 minutes in which the cameras are turned off**  *Ok, let’s turn on the camera again. Does everyone have a specific situation in mind? Or do you need more time? Now you have your situation in mind, I have a set of questions for you about it. You will be invited to share your answers with the group. But if you prefer to answer privately for some reason, feel free to type your answer in the private chat to moderator 2 (Name of the 2^nd^ Moderator)* | | | | **5** |
| 1c | **AIM**  In this part we will investigate the characteristics of the events and trigger sounds they remember. | **STAGE 1 QUESTIONS ABOUT THE EVENT**  *We will now go over some questions. Can you shortly describe the situation (the environment, the trigger sounds, people, circumstances, the time etc.)? Who would like to start?*  **If some people do not answer:** *Does anyone have any other situations they had in mind?*  **When everyone/most people answer, ask for more details:**   - *Where were you when you experienced this situation?* - *With whom were you?* - *In what mood were you prior to the situation?* - *What sound bothered you?* - *How did the situation end?* | | | | **20** |
| **2^ND^ SET OF QUESTIONS: EMOTIONS TOWARDS STIMULI & EVENTS** | | | | | | |
| 2a | **AIM**  In this part again, we want them to imagine how it felt like to be in this situation so we can get an insight on their feelings. | **STAGE 2 THINKING ABOUT EMOTIONS**  *Now, we will go over the emotions you definitely or maybe felt during this situation. What were you feeling, which emotions were definitely or maybe present? Take your time to come u up with an answer, I propose we again take 1-2 minutes to think about it. It can be helpful to turn off the camera, close your eyes, try to imagine yourself in the situation again. That might help you identifying what emotions or feelings were present. For instance, you can imagine, what you’ve felt in your body, and where you’ve felt it. Or maybe the thoughts you had at that time etc.*  **A thinking break for 2 minutes in which the cameras are turned off** | | | | **2** |
| 2b | **AIM**  In this part we want to find out how it felt to experience these situations. We want to get an insight into the emotions that were maybe or definitely present. | **STAGE 2 QUESTIONS ABOUT THE EMOTIONS**  *Does anyone like to start with sharing what they were feeling?*  ***If some people did not answer:*** *Is there anyone else who would like to add something to this? Maybe different feelings?*  **If everyone answered or no one has anything to add move to 2c**  **Moderator 2 registers notes on ‘Registration Form …’**  **Observer registers notes on..** | | | | **15** |
| 2c | **AIM**  In this part, we will gain information about the top 3 emotions for each of the participants. | **After the open question about feelings that were described, show the slide 1**: *This slide contains feelings that you described, and a set of potential other feelings. Please have a look at them and try to select the words that best express what you experienced in the situation. When you are ready with thinking, you can try to come up with a top 3 from the list you can see on the slides. You can also add something extra if it is not already mentioned on the list. When you are ready please write down your top 3 and share it in the group chat, you can also share it with the moderator in the private chat.*  **Moderator and the second moderator confirm that they got the answers from everyone. Now they can continue to the discussion questions in 2d** | | | | **10** |
| 2d | **AIM**  This part aims to get a more detailed insight into the emotions | *So, now that we thought about some of the emotions you experienced, I would like to ask you some other questions:*   - *What did you notice in your body? Where in your body did you notice the sensation?* - *What thoughts/ideas/expectations/memories went through your mind?* - *What did you do? feel like doing? What urges did you have? What did you actually do?*   **Moderator 2 registers the notes on Registration Form. The emotions and feelings will be added to our preparatory slides if they are not listed yet. The slides will be on google slides so every moderator can see/edit them as necessary.** | | | | **20** |
| 2e | **AIM**  To get quantitative data on the answers to our questions in the discussions | **STAGE 2 POLL**  *Now we would like to ask you to keep these discussions in mind and answer the following polls. You can select up to three items in each of the polls. You will see the poll on your screen. If there are any technical difficulties please raise your hand or write to the moderator in the private chat. Firstly, “What did you feel like doing?” show the poll* **(slide 2).** *Secondly, “What did you actually do?” show the poll* (**slide 3**)*. Thirdly, “What did you feel in your body and where?” show the poll* **(slide 4)***. Finally, “What thoughts did you have?” show the poll* **(slide 5)***.* | | | | **10** |
| **10 MINUTE BREAK** | | | | | | |
| **3^RD^ SET OF QUESTIONS: THE MEANING BEHIND THE EMOTIONS & REACTIONS** | | | | | | |
| 3a | **AIM**  In this last section we want to get insight into the meanings of the emotions towards the sounds. We want to know what it means for the participants to be in the situations they’ve described previously. | **STAGE 3 THINKING ABOUT THE MEANING**  *Now we will discuss the meaning that might possibly underly the sound for you personally. So, what we like you think about, is what did this situation potentially meant to you in order to trigger this feelings and reactions?* | | | | **3** |
| 3b |  | **STAGE 3 QUESTIONS ABOUT THE MEANING**  **Now the moderator will go in depth for more information.**   - *What does the situation / the sound mean to you that it makes you feel the way you described? Does anyone want to give it a try answering this?* - *What is specific about the sound that makes you react and feel the way you did, why do other sounds in other situations do not trigger these feelings or reactions? Is there a difference in meaning or significance?* - *What does the trigger sound mean to you?* - *What should happen to make the sound or the situation bearable to you?* - *Is the sound associated with certain individuals? If they answer yes: Around whom? What is different in hearing the sounds coming from someone else compared to that specific person. What feels different? Why is it more bearable if person x generates the sound compared to person y?* - *What is the worst that could happen in this situation?* | | | | **25** |
| 3c | **AIM**  To get quantitative data on the answers to our questions in the discussions | **After these open questions about meaning, we will show the slide 6 with possible meanings, and say:**  *This slide contains meanings that were shared / described in the group, and a set of other potential meanings. Please have a look at them, and try to select the meaning that matches best with the situation you have in mind. This meaning can be different for every person, so try not to let your answer influenced by the answers of others. There are no right or wrong answers! Try to come up with a top 3. You can send them to the moderator in a private chat.* | | | | **15** |
| 4a | **Closing of the meeting**  *We would like to thank all of you for your participation and your valuable contribution.*  *We would like to ask you to fill out a post-discussion questionnaire which you will receive in your email. It will probably take around 10-15 minutes to fill out. If you have any further questions or comments, please do not hesitate to contact us or stay a little longer in the discussion so we can go over them. You can contact with the researchers using the contact information on the informed consent form. And if you do not have any further comments or questions, we would like to offer you a small thank you gift, which you will find in your email after the discussion groups.*  *And thanks again everyone for participating, if you don’t have any further questions you can leave the meeting. Have a nice day/evening.* | | | | | **5** |
| 5 | **ONLINE POSTTEST PHASE (± 1 week before the test)** | | | | |  |
| 5a | After the participation in the discussions:   1. Participants will receive a link to a post discussion questionnaire via Qualtrics 2. After the completion of the questionnaire, the researchers will finalize their data entry 3. The participants will receive a thank you email with a link for an online gift voucher | | | | | **0** |

*The lines of the moderators are written in italics.

*The exact file names and doodle and Qualtrics links will be added to the documents as soon as they are ready.
