# Supplementary material for: Understanding the misophonic experience: a mixed method study
Source: Front Psychol. 2025 Feb 5;16:1493676. doi: 10.3389/fpsyg.2025.1493676 (PMC11835947; doi:10.3389/fpsyg.2025.1493676)
Supplement: Supplementary file 2 [file Table_2.DOCX]

**SUPPLEMENTARY MATERIALS**

**Supplementary Table 1.**

*Correlation matrix between the selected rating items (meanings)*

|  | Intrusion | Trapped in a Situation | Lack of Control | Lack of Autonomy | Violation |
| --- | --- | --- | --- | --- | --- |
| Intrusion |  |  |  |  |  |
| Trapped in a Situation | *.55* |  |  |  |  |
| Lack of Control | *.45* | *.6* |  |  |  |
| Lack of Autonomy | *.39* | *.43* | *.47* |  |  |
| Violation | *.48* | *.46* | *.42* | *.56* |  |
| Offence | *.28* | *.23* | *.31* | *.42* | *.44* |
|  |  |  |  |  |  |

*Note.* All correlations reported in the table were significant at *p < 0.001.*

**Supplementary Table 2.**

*Correlation between A-MISO-S scores and each rating.*

|  | **1** | **2** | **3** | **4** | **5** | **6** | **7** | **8** | **9** | **10** | **11** | **12** | **13** | **14** | **15** | **16** | **17** | **18** | **19** |
| --- | --- | --- | --- | --- | --- | --- | --- | --- | --- | --- | --- | --- | --- | --- | --- | --- | --- | --- | --- |
| **1.** A-Miso-S Score |  |  |  |  |  |  |  |  |  |  |  |  |  |  |  |  |  |  |  |
| **2.**Relax | -.37* |  |  |  |  |  |  |  |  |  |  |  |  |  |  |  |  |  |  |
| **3.**Offence | .29* | -.11 |  |  |  |  |  |  |  |  |  |  |  |  |  |  |  |  |  |
| **4.**Need for escape | .41* | -.38* | .28* |  |  |  |  |  |  |  |  |  |  |  |  |  |  |  |  |
| **5.**Anger | .52* | -39* | .4* | .57* |  |  |  |  |  |  |  |  |  |  |  |  |  |  |  |
| **6.**Intrusion | .46* | -.38* | .28* | .49* | .61* |  |  |  |  |  |  |  |  |  |  |  |  |  |  |
| **7.**Violation | .45* | -.24* | .44* | .43* | .57* | .48* |  |  |  |  |  |  |  |  |  |  |  |  |  |
| **8.**Lack of autonomy | .42* | -.13 | .41* | .35* | .44* | .38* | .56* |  |  |  |  |  |  |  |  |  |  |  |  |
| **9.**Calm | -.33* | -.68* | -.12 | -.36* | -.43* | -.37* | -.26* | -.15 |  |  |  |  |  |  |  |  |  |  |  |
| **10.**Defensive anger | .51* | -.35* | .36* | .47* | .76* | .54* | .57* | .45* | -.33* |  |  |  |  |  |  |  |  |  |  |
| **11.**Happy and excited | -.16* | -.53* | .02 | -.16* | -.19* | -.18* | -.06 | .02 | .51* | -.16* |  |  |  |  |  |  |  |  |  |
| **12.**Trapped in a situation | .47* | -32* | .23* | .5* | -.53* | .55* | .46* | .43* | -.28* | .5* | .08 |  |  |  |  |  |  |  |  |
| **13.**Guilty | .32* | -.14 | .22* | .29* | .33* | .23* | .34* | .37* | -.15* | .36* | .01 | .32* |  |  |  |  |  |  |  |
| **14.**Disgust | .27* | -.33* | .31* | .44* | .5* | .46* | .39* | .23* | -.27* | .4* | -.24* | .44* | .25* |  |  |  |  |  |  |
| **15.**Afraid | .29* | -.06 | .29* | .25* | .27* | .23* | .39* | .44* | -.08 | .28* | .08 | .35* | .49* | .19* |  |  |  |  |  |
| **16.**Avoiding the sounds | .4* | -.5* | .17* | .52* | .56* | .59* | .34* | .28* | -.43* | .43* | -.28* | *.56 | .2* | .55* | .17* |  |  |  |  |
| **17.**Peaceful | -.21* | .57* | -.11 | -.38* | -.28* | -.33* | -.15 | -.04 | .63* | -.26* | .53* | -.17* | -.03 | -.22* | .04 | -.33* |  |  |  |
| **18.**Lack of control | .46* | -.22* | .31* | .4* | .53* | .45* | .42* | .46* | -.24* | .44* | -.03 | .59* | .32* | .33* | .34* | .44* | -.13 |  |  |
| **19.**Enthusiastic | -.16* | .52* | .03 | -.14 | -.17 | -.22 | -.08 | .04 | .51* | -.17* | .65* | -.08 | .03 | -.17* | .1 | -.22* | .63* | -.05 |  |
| **20.**Sad | .44* | .12 | .4* | -.3* | .42* | .31* | .49* | .51* | -.15* | .4* | .01 | .43* | .39* | .27* | .47* | .26* | -.1 | .4* | .09 |

** p < 0.001*
